# Supplementary material for: Polymer-Infiltrated Metal–Organic Frameworks for Thin-Film Composite Mixed-Matrix Membranes with High Gas Separation Properties
Source: Membranes (Basel). 2023 Feb 28;13(3):287. doi: 10.3390/membranes13030287 (PMC10053294; doi:10.3390/membranes13030287)
Supplement: Supplementary file 1 [file membranes-13-00287-s001.zip › membranes-2217286-supplementary.pdf]

membranes

Supplementary Materials

Polymer-Infiltrated Metal–Organic Frameworks for Thin-Film Composite Mixed-Matrix Membranes with High Gas Separation Properties

Hyo Jun Min [1,†], Min-Bum Kim [1,2,†], Youn-Sang Bae [1], Praveen K. Thallapally [2], Jae Hun Lee [3,*] and Jong Hak Kim [1,*]

1 Department of Chemical and Biomolecular Engineering, Yonsei University, 50 Yonsei-ro, Seodaemun-gu, Seoul 03722, Republic of Korea
2 Pacific Northwest National Laboratory, 902 Battelle Boulevard, Richland, WA 99352, USA
3 Hydrogen Research Department, Korea Institute of Energy Research, 152 Gajeong-ro, Yuseong-gu, Daejeon 34129, Republic of Korea
* Correspondence: jhlee@kier.re.kr (J.H.L.); jonghak@yonsei.ac.kr (J.H.K.)
† These authors contributed equally to this work.

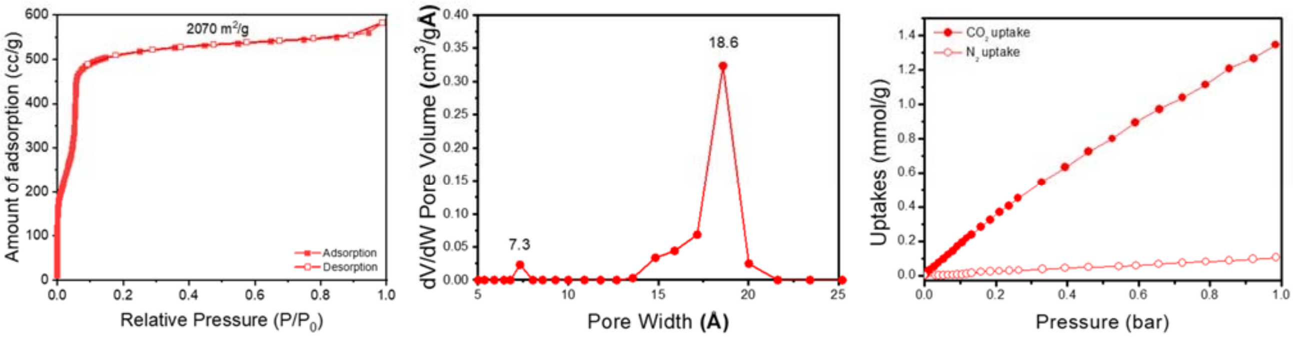

(a) (b) (c)

Figure S1. (a) BET surface area and (**b**) pore size distribution from N_2 isotherm at 77K and (**c**) CO_2 and N_2 adsorption isotherms of MOF-808 at 303K.

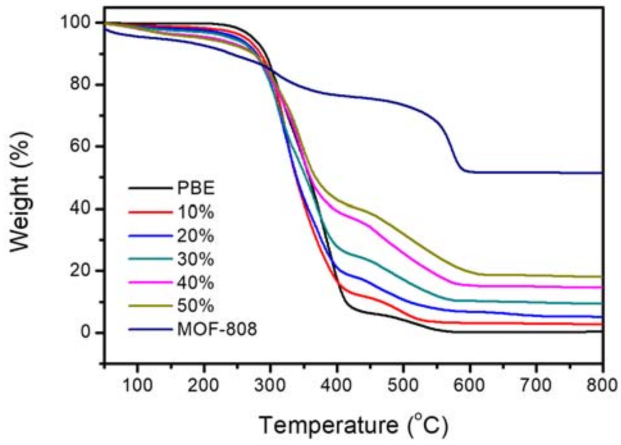

Figure S2. TGA curves of neat PBE and PBE/MOF-808 MMMs with different MOF-808 contents.

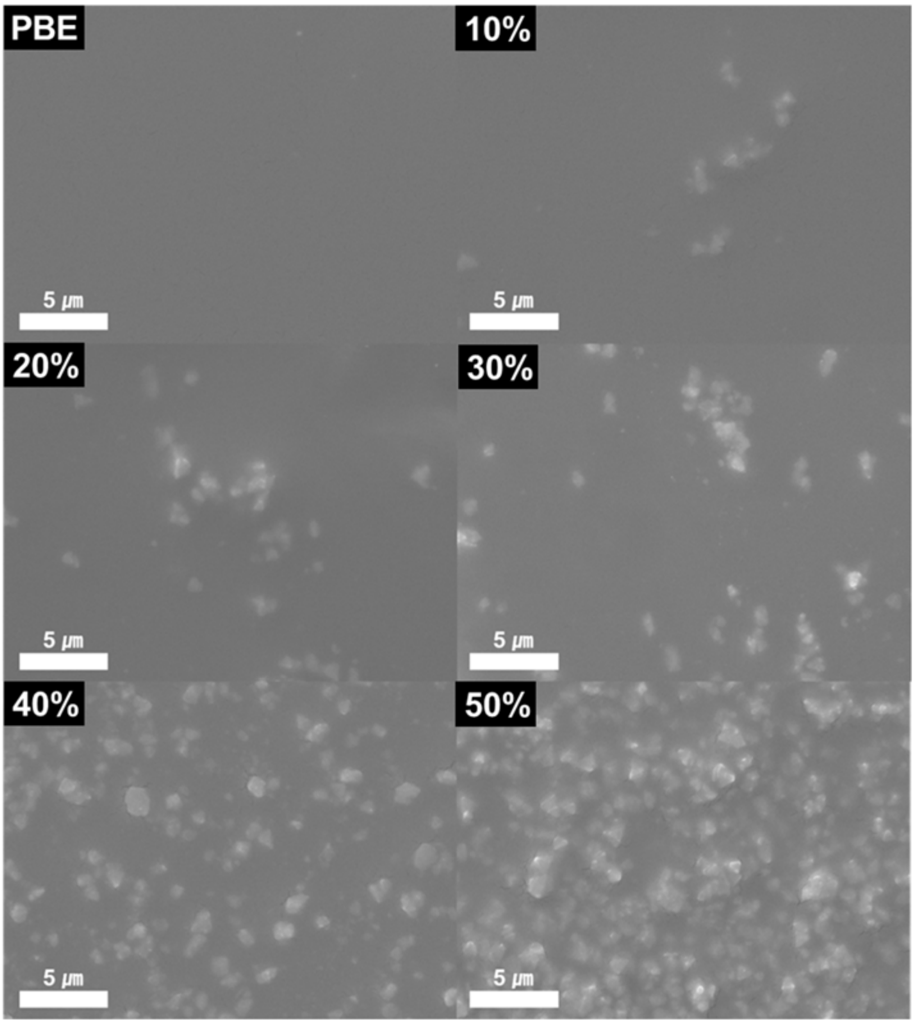

Figure S3. Surface SEM images of neat PBE and PBE/MOF-808 TFC-MMMs with different MOF-808 contents.
